# *PhagoSight*: An Open-Source MATLAB® Package for the Analysis of Fluorescent Neutrophil and Macrophage Migration in a Zebrafish Model

**DOI:** 10.1371/journal.pone.0072636

**Published:** 2013-08-30

**Authors:** Katherine M. Henry, Luke Pase, Carlos Fernando Ramos-Lopez, Graham J. Lieschke, Stephen A. Renshaw, Constantino Carlos Reyes-Aldasoro

**Affiliations:** 1 MRC Centre for Developmental and Biomedical Genetics, University of Sheffield, Sheffield, United Kingdom; 2 Cancer and Haematology Division, Walter and Eliza Hall Institute of Medical Research, Parkville, Australia; 3 Institute of Toxicology and Genetics, Karlsruhe Institute of Technology (KIT), Eggenstein-Leopoldshafen, Germany; 4 Biomedical Engineering Research Group, University of Sussex, Falmer, United Kingdom; 5 Department of Medical Biology, University of Melbourne, Parkville, Australia; 6 Australian Regenerative Medicine Institute, Monash University, Clayton, Australia; 7 Information Engineering and Medical Imaging Group, City University London, London, United Kingdom; University of Birmingham, United Kingdom

## Abstract

Neutrophil migration in zebrafish larvae is increasingly used as a model to study the response of these leukocytes to different determinants of the cellular inflammatory response. However, it remains challenging to extract comprehensive information describing the behaviour of neutrophils from the multi-dimensional data sets acquired with widefield or confocal microscopes. Here, we describe *PhagoSight*, an open-source software package for the segmentation, tracking and visualisation of migrating phagocytes in three dimensions. The algorithms in *PhagoSight* extract a large number of measurements that summarise the behaviour of neutrophils, but that could potentially be applied to any moving fluorescent cells. To derive a useful panel of variables quantifying aspects of neutrophil migratory behaviour, and to demonstrate the utility of *PhagoSight*, we evaluated changes in the volume of migrating neutrophils. Cell volume increased as neutrophils migrated towards the wound region of injured zebrafish. *PhagoSight* is openly available as MATLAB® m-files under the GNU General Public License. Synthetic data sets and a comprehensive user manual are available from http://www.phagosight.org.

## Introduction

Multiphoton and confocal fluorescence microscopy, which allow 3D imaging of specimens *in vivo* with high spatial and temporal resolution, have been widely adopted in the Life Sciences [1], [2] for varied applications including: the observation of microvascular permeability [3]; the assessment of mitochondrial function [4], [5]; examining tumour microcirculation [6] and angiogenesis [7]; and the observation of neutrophil apoptosis and migration [8]. Confocal and multiphoton microscopes capture the intensity value (*i*) at a specific three dimensional location (*x,y,z*). This intensity is related to the photons collected at the detector, which are in turn emitted by fluorescent substances in the sample, in response to excitation at specific frequencies (*f*). As the observation is repeated in time (*t*), the data become a 5-dimensional matrix *i(x,y,z,f,t)*. Thus, a single experiment can easily generate many gigabytes of information, presenting a significant challenge for data transfer, storage and processing.

Whilst the considerable cost of modern microscopes and the availability of skilled operators has been a limitation for their use in the past, they are now common-place in academic centres, either in dedicated laboratories or as part of core facilities. However, acquisition is only the start of the process, and expertise has lagged for the processing, segmenting, quantification, analysis and interpretation of the wealth of information contained in the very large data sets produced by these microscopes. In many cases, data is acquired at a faster rate than it can be processed and many laboratories require human expert users that spend many hours examining visually the acquired images and videos. Inadvertently, technological advances have shifted the bottleneck of these biomedical experiments from the data generation to the data processing [9], [10]. This is particularly acute where biological models are amenable to manipulation and imaging.

**Figure 1:**
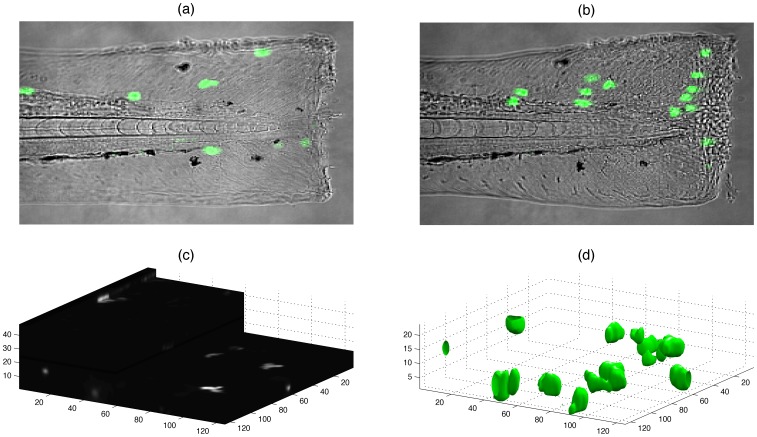
Visualisation of neutrophils in zebrafish. (a,b) Fluorescent neutrophils (bright uniform regions) migrate towards the site of injury (right) in the tail-fin of a zebrafish larva (differential interference contrast (DIC)) at 3 dpf. (c) One time point of 48 slices each of 1024×1024 pixels. (d) Neutrophils rendered as 3D surfaces.

Inflammation is a process critical to life itself, without which multicellular animals could not protect themselves against the threat of competing unicellular microorganisms or tissue injury. Zebrafish larvae have emerged as a key model organism for inflammation studies, with a unique combination of advantages over other model systems for the detailed study of inflammation biology *in vivo.* Understanding cell migration and interaction is an important part of understanding how immune cells behave during all phases of inflammation *in vivo*. The optical transparency of zebrafish allows visualisation of physiological and pathological processes *in vivo*. Genetic manipulations can be easily performed, both to genetically manipulate the inflammatory response and to label individual cell populations with fluorescent markers *in vivo* [11]. These cell populations can then be observed in high temporal and spatial resolution during inflammation, using multiphoton and confocal microscopy (Fig. 1).

Many publications related to neutrophils in zebrafish and other models rely on manual processing by an expert [12], [13], [14], [15]. Cell tracking is sometimes performed with commercial systems linked to the acquisition hardware such as Volocity® (Perkin Elmer™, USA) [16], [17] or Imaris® (Bitplane™, Switzerland) [18], [19]. Commercial software is expensive and in some cases lags behind the demands of researchers in the field, as companies develop tools that can be used for a wide variety of experiments and only develop specific algorithms when justified by a large demand from the scientific community. Some of the current limitations of the commercial packages for the analysis of neutrophils in zebrafish are related to the segmentation, which is sometimes performed with a single threshold, which introduces artefacts, or watersheds [20] that are susceptible to over-segmentation problems [21].

Alternatively, researchers often employ programming tools such as MATLAB® (Mathworks™, USA) and the similar freeware options Scilab and Octave, Mathematica® (Wolfram Research™, USA), R, Python or Java™ to develop their own algorithms for specialised analytical purposes e.g. a leukocyte tracking and statistical analysis framework developed in R and Python is presented in [22], [23] and shape-based tracking of cells is presented in [24]. A third option is specialised tracking plug-ins of ImageJ [25] like MTrackJ [26], Particle Tracker [27], TrackMATE [28] or the complete open package ICY [29]. A limitation of both commercial and specialised software is that segmentation algorithms are specific to the visual appearance of the cells and the variation of imaging protocols requires modification of the algorithms [30].

The segmentation of fluorescent phagocytes is complicated due to the complex variations of the shape: a single cell that is active and has expanded pseudopods can have a range of intensities and may be artificially segmented into several unconnected objects. When two cells are close to each other, the gap between can be too small to be distinguished and then two cells can be considered to be a single cell.

To address the challenging task of analysing the motion and shape tracking of neutrophils in zebrafish, we have developed *PhagoSight,* a series of algorithms in the MATLAB® programming environment. The package provides semi-automated algorithms that read and transform large data sets into MATLAB® format, segment and track phagocytes, and provide a large number of quantitative measurements from which users are able to analyse the behaviour of their data sets. The package performs many pre- and post-processing steps: intensity thresholds are pre-selected based on Otsu’s algorithm [31] which the user then can verify manually if desired. Temporal variation of intensity is analysed as cells that disappear from their tracks and then re-appear a few points later. Collisions of cells are analysed by measuring the volume of cells in time and splitting cells whose volume increases considerably. Finally, as the lack of proofreading and editing tools has been one of the main barriers in adopting automated and semi-automated methods [32] we provide such tools, through which users can evaluate the output of algorithms and correct mistakes that can be visually detected.

A series of synthetic data sets are also provided as a means to evaluate the robustness of the algorithms under different conditions. All the algorithms are released in an open policy under the GNU General Public License, with three main objectives. Firstly, it allows other researchers to use the algorithms and advance their research on neutrophils in zebrafish. Secondly, it allows other researchers to adapt and modify the software to suit their experiments. In turn, this may produce refined algorithms and routines that will be incorporated to the software package that will be improved iteratively. Thirdly, open access software allows independent replication and verification. As experiments become more complex, produce larger volumes of data and rely on proprietary software or code, the replication and verification becomes increasingly difficult [33]. It should be noted that the tracking algorithms that we present are *a posteriori* tools to be applied to a sequence of images or volumes, and not intended to be used as ‘on-the-ﬂy’ tracking techniques [34].

The algorithms of *PhagoSight* were tested on synthetic and biological data sets in order to determine the reliability and robustness of the algorithms against noise and sensitivity to the input parameters. Moreover, we use these approaches to test the hypothesis that neutrophils increase their volume as they migrate towards the wound region in a zebrafish model of inflammation.

## Experimental Procedures and Data Acquisition

### a. Zebrafish

Zebrafish were maintained according to standard protocols [35]. The biological data sets were acquired from transgenic *Tg(mpx:eGFP)i114* zebrafish larvae in which neutrophils specifically express Green Fluorescent Protein (GFP) [11]. Tail-fin transection was performed on zebrafish larvae at 3 days post fertilisation (dpf) as previously described [11]. Zebrafish larvae were mounted in low melting point agarose (Sigma) immediately prior to imaging. Multiple larvae were imaged simultaneously using a moving stage. Temperature was maintained by environmental air conditioning at 24°C.

### b. Image Acquisition

To assess neutrophil volume, 301 time points of 1000×1000 pixels in 32 slices in the GFP channel (exposure 40 ms) at 5 µm step size and 1 brightfield reference image were captured using an UltraVIEWVoX spinning disk confocal microscope (PerkinElmer Life and Analytical Sciences), scanning once per slice, with a 20×objective NA 0.75 for six injured larvae. Data were acquired using Volocity® 6.0.1 at a rate of 120 time points per hour for 2.5 hours beginning at approximately 0.7 hours post injury. Each 3D stack took approximately 1.4 seconds to acquire. The increased resolution was used to obtain a more reliable measurement of the volume in an area directly anterior to the wound region. Multiple TIFF files were exported from Volocity® and were read and subsequently analysed using *PhagoSight*.

### c. Statistics

Statistical analysis was performed using Prism™ 5.0 (Graphpad Software Inc., San Diego, CA). Differences were considered significant at *P*<0.05. Measurements obtained with *PhagoSight* are presented as the mean ± standard error of the mean unless otherwise stated. Linear regression analysis was performed using Prism™ 5.0.

### d. Ethical Considerations

Zebrafish studies were performed in accordance with UK Home Office legislation. UK law requires that, where possible, experiments are performed on animals not protected under the Animals (Scientific Procedures) Act. All experiments were performed on unprotected embryos, <5.2 dpf.

## Design and Implementation

*PhagoSight* has been developed taking into account that the end-users may not be expert MATLAB® programmers and therefore combines graphical user interfaces (GUI) and the use of written commands. The input data is stored in a series of folders, one for each time point of observation and it can be either of the following formats: (a) one image for every slice of a 3D stack, (b) one 3D Tiff image or (c) a MATLAB® file which contains a 3D matrix. *PhagoSight* stores the following intermediate results: original images in MATLAB® format, reduced images, segmented and labelled images and the final results, which are referred to by the term “*handles*”, in separate folders with the original name and an identifier: e.g. *images_mat_Or, images_mat_Re, images_mat_La* and *images_mat_Ha,* are created when the input data is in a folder called *images*.

### a. Pre-processing of the Data

The first pre-processing step is a reduction of the size of data through smoothing and subsampling, which is a common technique to reduce the computational complexity (number of operations and time required to process) of the processing and to reduce the noise of the data. The reduction is obtained by averaging groups of four contiguous voxels on each z-slice; their mean value is then assigned to a voxel in a new image, which will have a reduced number of rows and columns, the number of z-slices remains unchanged. Thus the signal to noise ratio (quality of the image) is improved at the expense of a reduced spatial resolution.

The structure of the input data from microscopes can vary considerably; it is possible to acquire data in several fluorescent or differential interference contrast (DIC) channels, which are then saved at different positions of a single stack of images. In the second pre-processing step, *PhagoSight* analyses the intensity histograms of each slice of the data and generates an initial estimate of the distribution of the channels. This is based on the observation that fluorescent channels concentrate the majority of the pixels or voxels at low intensity levels, whilst DIC channels have a higher concentration of pixels in the middle intensities and lower numbers of pixels at either end of the intensity range. The user can verify the distribution of the channels through a GUI with the histograms and accept or modify the initial estimate.

**Figure 2:**
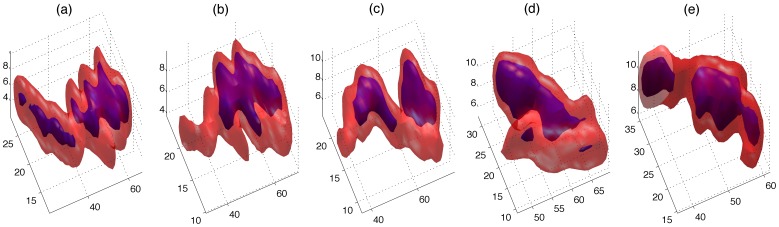
Hysteresis thresholding segmentation of a single neutrophil at five time points. The region described by low threshold (bright) will contain one or more regions described by the high threshold (dark); if segmented solely with a single high threshold several unconnected regions would arise. A single low threshold would in turn produce more regions of low intensity not shown here.

Since neutrophils can stretch and change their shape considerably between time points, it is very important to use a segmentation procedure that (a) does not include background as cells and (b) does not over-segment a single cell into several disjointed objects. The third pre-processing step is the definition of the intensity thresholds with double *hysteresis* threshold inspired by the *Schmitt trigger* [36]: voxels below a lower threshold are classified as background, and those above a higher threshold are classified as neutrophils. The remaining voxels between these two levels are then classified as neutrophils if they are in contact with voxels above the high threshold, or as background otherwise. Both thresholds are automatically determined using Otsu’s algorithm [31]. Fig. 2 shows the segmentation of a single neutrophil with two thresholds. Since the selection of thresholds can have an impact on the results, a GUI allows the user to verify the accuracy of these threshold levels.

**Figure 3:**
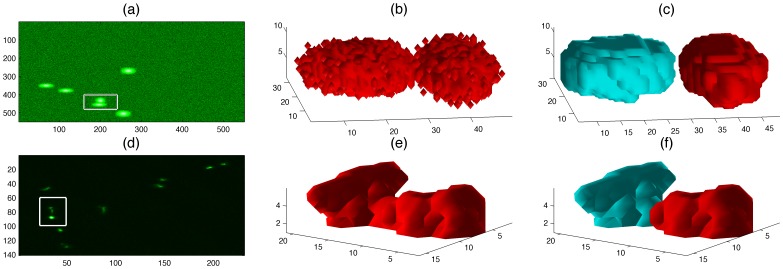
Segmentation of large objects in synthetic (top row) and real (bottom row) data sets. (a,d) One slice of a 3D stack where one object (box) was considered as an outlier due to its volume. (b,e) A three-dimensional rendering of the object indicates that it is formed by two neutrophils that collided. (c,f) Two new objects after the segmentation.

The final and important pre-processing step analyses the volumes of the neutrophils after the segmentation procedure is complete. The algorithm obtains the distribution of the volumes of all neutrophils at all time points and calculates mean and standard deviation with the objective of detecting outliers in the distribution. A neutrophil whose volume exceeds mean +3× standard deviation of the distribution is assumed to be formed by two neutrophils that are too close to be separated by the segmentation algorithm. Those objects are split into two disjointed objects following a sequential erosion (removal of the voxels at the surface of the object) of the object until the voxels that were bridging the two objects are removed (Fig. 3).

### b. Tracking Algorithm

Following segmentation, individual neutrophils are assigned unique labels. The corresponding results are stored in separate folders with the extension *mat_La*. The tracking process consists of linking a neutrophil at time *t*, with the same neutrophil at time *t+1*. This correlation process is trivial when only one cell is present, but it may be very complicated with more than two neutrophils. To simplify the analysis there are studies that restrict the input data to those cases that contain a single neutrophil [37], [38], [39], while others restrict the conditions of movement so that neutrophils overlap within time points [23], [40], that is, they only analyse data sets with slow movement. Other studies evaluate visually the distinguishable condition of each cell [41] and only process those that satisfy a human operator. *PhagoSight* does not make any assumptions on the velocities, conditions or number of neutrophils. The fluorescently-labelled neutrophils are tracked with a model-based tracking algorithm [42] adapted from the keyhole tracking algorithm presented in [43]. The algorithm links the objects in contiguous time points to form the tracks by means of a keyhole model, which predicts the most probable landing position of a neutrophil at time *t+1* (which we called “child”), from the position in times *t* (the “parent”), and *t−1* (the “grandparent”). The most probable step for a neutrophil that is moving from time *t−1* to time *t*, is to follow the direction of the previous steps with the same velocity to time *t+1*. Assuming that a child (neutrophil at time *t+1*) would move with similar direction and velocity as its parent (neutrophil at time *t*), its landing position can be predicted. Of course, this would not cover changes in speed or turns or random walk-like movements. Two regions of probability where the child neutrophil is most likely to land are therefore defined: a narrow wedge (60° wide) oriented towards the predicted landing position, for straight-moving displacements, and a truncated circle (300°) that complements the wedge, for random-moving displacements, which together resemble a keyhole. The size of the keyhole at *t+1* is determined by the distance between times *t−1* and *t*. All segmented neutrophils are examined for possible parent-child relationships within keyholes; when there is more than one possible relationship, the closest to the predicted landing site is assigned.

### c. Post-processing

The post­processing steps increase the reliability of the tracks produced through several steps. Firstly, the same keyhole model is used to analyse the movement backwards. That is, the same keyhole model uses child (*t+1*) and grandchild (*t+2*) to generate a keyhole at time (*t*). If the neutrophil of a previous time point lands inside the keyhole, it remains as part of the track, otherwise it is removed. This is especially important for the first link of a track when there is no previous history of movements. Secondly, consecutive labelled points are compared to detect cells that are present at time *t−1*, absent at time *t*, and present at *t+1*, within a certain small region of interest. We consider these cases to be due to cells with low intensity, which due to slight variation in fluorescence over time, are too faint to appear in all time points. For those cases of disappearing cells, an artificial cell is created at time point *t* as an interpolation of cells at times *t−1* and *t+1*. A subsequent tracking procedure links the two previously disconnected tracks into a single track.

An important post-processing step is to detect collisions between neutrophils since not all cells that collide can be detected as outliers due to their volume. In some cases, when two small neutrophils collide, their volume may not be much larger than a single large neutrophil. To detect two neutrophils that travel towards each other until they are too close to be distinguished as two separate cells, we follow two rules: (a) the volume of a given neutrophil increases considerably and (b) a neighbouring track terminates in the previous time point. In the same way, after a collision, cells may seem to divide. The rules to detect these divisions are the inverse: (a) a decrease in neutrophil volume and (b) one new track starts on the current time point. Unlike in the case of pre-processing, where large volumes are split into two disjointed objects, the collision could involve more than two neutrophils that form one large fluorescent cell. The segmentation of the merged cells is performed with the watershed transformation. The watershed transformation partitions the images into catchment basins or regions of influence of the regional minima. The boundaries between the catchment regions are called watersheds, and are used to segment into the cells that collided. Once those cells are divided, new tracking is performed.

### d. Directional Analysis and Measurement Generation

**Figure 4:**
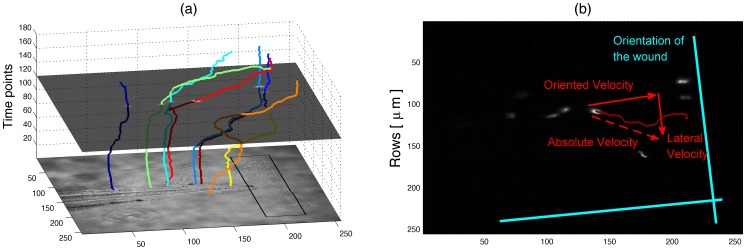
Orientation of the movement based on the artificial wound region. (a) Three-dimensional plot of the tracks with time as the vertical axis. The DIC image of the fish is presented as a horizontal plane at time 0 and one fluorescent slice is shown at time 120. The black square over the DIC corresponds to the artificial wound region. (b) Description of the absolute, oriented and lateral neutrophil velocities with respect to the axis defined according to a manual delineation of the wound region.

To analyse the directionality of the movement and its nature (fast/slow, uniform/varying velocity, direct/meandering, etc.) a series of measurements based on the tracks are calculated. As such, it is necessary to determine an orientation framework as the tail of the zebrafish can have any orientation within the acquired images. The tail-fin transection, a wound towards which the phagocytes are attracted, is performed at the opposite side of the head of the fish and a wound region is created. We identify manually a region of interest, which corresponds either to the region of the wound, or the corresponding region in an uninjured control larva. We term this the “artificial wound region”. Fig. 4a shows this artificial wound region as a black rectangle overlaid on the DIC image of the tail. This wound region is then used to generate a rotation of the axis with the movement towards the wound as one coordinate axis (*c*) and lateral movements as the other axis (*r*) (Fig. 4b). Each movement is analysed as a vectorial projection towards a line perpendicular to the wound. The components of the movement towards and parallel to the wound are considered the *oriented distance* and *lateral distance* respectively, and are used to calculate *oriented* and *lateral velocities*. The absolute velocity corresponds to the vector sum of the other two components. The oriented movements can be further analysed in terms of how effective they are in moving towards the wound: assigning +1 for movement parallel to the main orientation towards the wound, 0 for movement perpendicular to the main orientation, *−*1 for perpendicular moving away from the wound and between (*−*1,+1) for any other orientation. In this way, not only the absolute velocity (how fast the cell moved), but also the oriented velocity (how fast it moved towards the wound), effective movements (ratio of displacements between points that move more than a particular threshold) are observed. In a recent publication, *PhagoSight* was used to compare the speeds of neutrophils in the presence or absence of Cxcl8 [44].

It is of great biological interest to observe the motion of neutrophils towards areas of inflammation. The manually delineated *artificial wound region* described above allows the determination of several important behaviours, such as the number of neutrophils entering this region and the time they stay within it. It is important to notice that the wound region is manually drawn by the user and thus is subject to intra- and inter-observer variability. Therefore, the size of the wound region can influence measurements such as the arrival of a neutrophil at the wound. When comparing several experiments, we recommend either generating wound regions of the same size, say 100, 120, 150 pixels wide, or performing a sensitivity analysis with wound regions of varying sizes and observing the impact on the measurements. Of course, the size of the wound region will depend on the resolution of the camera, magnification of the microscope and the size of the tail relative to the image. Representative measurements that summarise the behaviour of each cell and/or the whole population of neutrophils in each zebrafish larva included in *PhagoSight* package are described in Table 1.

**Table 1:** Computationally derived measurements used to assess neutrophil migration following wounding.

Measurement	Definition
Velocity	Average absolute velocity per track
Oriented velocity	Average oriented velocity per Track
Lateral velocity	Average lateral velocity per track
Meandering index	Ratio of the shortest distance between two points relative to the distance that a neutrophil covers between those points
In-wound neutrophils	Number of neutrophils that reach the region designated as “wound region”
Forward ratio	Ratio of “number of displacements with effective velocity larger than 0.6” to the “total number of displacements”
In wound ratio	Ratio of “number of displacements inside the wound region” relative to “total number of displacements”
In wound ratio 2	Ratio of “number of displacements inside the wound region” relative to “number of displacements after a neutrophil reached the wound region”
Idle wound ratio	Ratio of “number of displacements with absolute velocity lower than a certain level” relative to “number of displacements inside the wound region”
Backward ratio	Ratio of “number of displacements with effective velocity larger than *−*0.6” to the “total number of displacements”
Leave wound ratio	Ratio of “number of displacements with effective velocity larger than *−*0.6” to the “number of displacements after the wound was reached”
Transiting wound neutrophils	Number of tracks that enter the wound and leave

### e. Output of the Algorithms

The tracking algorithm produces a series of 4D vectors (*x,y,z,t*) for each neutrophil. The tracks, and many other measurements, are stored in a single MATLAB® structure called *handles*. Structures are records that contain several values called fields; for example (*x,y,z*) coordinates, time points, etc. are stored within handles. The tracks are stored in two fields: *handles.nodeNetwork* and *handles.finalNetwork*. nodeNetwork contains the information of each segmented neutrophil; its (*x,y,z,t*) position and its parent-child relationship with several other measurements. finalNetwork is a matrix with one column for every track, the number of the segmented objects that belong to each track is stored in the columns.

### f. Proofreading and Editing Tools and user Manual

Algorithms can perform many tasks with consistency and speed above that of human observers. However, algorithms are not perfect and human observers have superb innate visual processing skills. Therefore, we provide a series of tools that allow users to evaluate the output of the segmentation and tracking process, and if necessary, to correct mistakes. We expect that these tools will lower the adoption barrier of those users who trust their own eyes more than automated solutions [32]. There are three editing functions: *delete, merge* and *break*. Delete simply removes a track from the analysis, and should be used with caution so as to not introduce unintentional bias into the analysis. Merge and break are closely related and may be necessary when either the track of a single neutrophil appears as two disjointed tracks or when a neutrophil collision occurs and the tracks follow the neutrophils incorrectly. In those cases, the tracks may be broken and merged according to the users’ visual criteria. Beltman et al. [45] suggest assigning different tracks to a single cell before and after a collision to avoid switching of tracks, and recognise that the splitting of tracks obscures the long-term behaviour of the cell. We consider that the manual breaking and merging of tracks, together with the use of cell volume when analysing the collisions will result in measurements that better reflect the long-term behaviour of cells.

The website http://www.phagosight.org contains a comprehensive user’s manual covering an introduction to MATLAB®, segmentation, tracking, data structure, visualisation tools and video generation. Synthetic data sets are also available.

### g. Visualisation Tools

*PhagoSight* provides several visualisation tools. We consider that three-dimensional plots show the kinetic behaviour of the neutrophils in several ways. Firstly, they display the general direction of movement of the cells, in our examples towards the site of injury in general. Secondly, the individual velocity is related to the slope of the lines, horizontal lines correspond to fast-moving cells and vertical ones, to slow or nearly stationary cells. Thirdly, they display how some cells migrate towards the wound (black line overlaid on the DIC in Fig. 4a) and, once there, remain static.

## Results

### a. Validation of the Algorithms with Synthetic and Biological Data Sets

**Figure 5:**
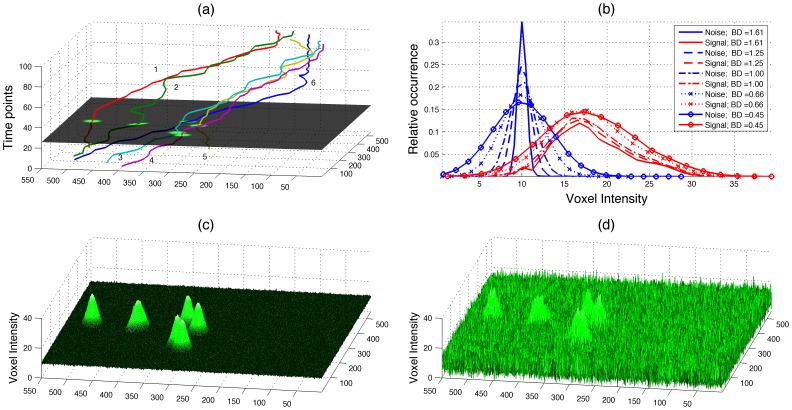
Description of the synthetic data sets. (a) One slice at t = 26 and the paths of six neutrophils shown as coloured lines. Vertical axis indicates time. (b) Five histograms for background (noise) and neutrophils (signal) for different levels of noise. The separability is indicated by the Bhattacharyya Distance (*BD*) values: highest *BD* corresponds to more separable classes (solid lines with no markers) and lowest *BD* corresponds to less separable classes (solid lines with circle markers). (c) One slice (*BD* = 1.61, SNR = 25.2 dB) shown as a mesh, intensity corresponds to the vertical axis. (d) One slice (*BD* = 0.45, SNR = 10.7 dB). The noise can be easily compared between the two data sets.

We validated the segmentation and tracking algorithms of *PhagoSight* with synthetic and real data sets. The synthetic 3D data sets spanned 98 time points with 11 slices of 275×275 pixels each. Six artificial neutrophils, modelled with anisotropic Gaussian shapes of different orientations, travelled along manually drawn paths that presented different conditions of tortuosity, times to activation and proximity to other neutrophils. An original noiseless data set was corrupted by adding white Gaussian noise of increasing variance to create five noisy data sets with increasing similarity between the neutrophils and the background (Fig. 5). The similarity was reflected by the decreasing values of the Bhattacharyya Distance (*BD*) [46], [47] (1.61, 1.25, 1, 0.66, 0.45) between the neutrophils and the background. The *BD* was calculated from the means and variances of the neutrophils and background in the following way [48]:
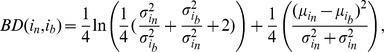
where 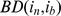 is the *BD* between the intensities of neutrophils 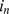 and background 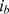, 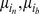 and 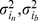 are their corresponding means and variances. The corresponding signal-to-noise ratios (SNR) were (25.2, 20.6, 17.4, 13.3, 10.7) dB. We calculated the SNR as 20 × logarithm base 10 of the ratio of root mean squared (RMS) pixel intensity of the neutrophils to the RMS pixel intensity of the background; the RMS was calculated as 
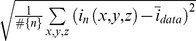
, where #{*n*} denotes the number of elements (neutrophils/background), 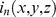 corresponds to the intensity of a given element and 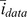 corresponds to the average intensity of the set. In addition to these data sets, *PhagoSight* also contains a series of synthetic data sets with irregular shapes that have been corrupted with Poisson and Gaussian noise, which is a better model of the noise that is associated with multiphoton and confocal microscopy [49], [50]. The irregular shapes were formed by overlapping an isotropic Gaussian, considered as the basic shape, and six isotropic Gaussians whose centres were shifted from the basic shape’s centre by a random distance in *x* and *y*. This combination created a more realistic neutrophil gold standard. Then, a combination of Poisson and Gaussian noise was added to the gold standard to create five data sets.

The real data sets corresponded to nine sets of fluorophore-based imaging leukocytes in zebrafish, one image in the fluorescent channel and one DIC image over 180 time points, with differences in neutrophil numbers (between 4 and 20), shapes, behaviour, and fish models (neutrophil-replete but myeloperoxidase-deficient mutant (durif) and wild-types [51]). The neutrophils were manually tracked during 180 time points to obtain a “gold standard”: each neutrophil was tracked by selecting its centroid at each time point with a custom-based MATLAB® interface.

We defined two measurements of accuracy: (a) distance from the centroids of the automatically tracked neutrophils to those of the gold standard, D_AG_, and (b) distance from the centroids of the gold standard neutrophils to those of the automatically tracked neutrophils, D_GA_. It was important to use both measurements, as there could be scenarios with good outcomes in one but not the other measurement. For instance, if the automatic tracking would detect just one very bright neutrophil and discard several faint ones due to a high threshold, D_AG_ would be small as long as the automatic track were close to the gold standard. However, D_GA_ would be large as there would be many tracks in the gold standard with no corresponding automatic tracks. The opposite case, a low threshold that would segment and track all neutrophils correctly, but also include noise and incorrectly track it and fail to distinguish colliding neutrophils, would result a low D_GA_ as the gold standard tracks were close to the automatic ones, but a high D_AG_, due to all the incorrect tracks.

**Figure 6:**
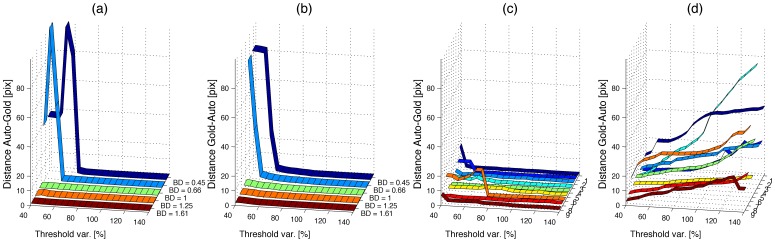
Validation of the algorithms with synthetic and real data sets. In all cases the sets were automatically tracked with *PhagoSight*; input thresholds were automatically determined and then modified from 40% to 140% of the original values to test the robustness against variation of that input parameter. (a) Distance from the automatically generated tracks to the gold standard (D_AG_) for the synthetic data set, *BD* corresponds to the Bhattacharyya distance between background and neutrophils. (b) Distance from the gold standard to the automatically generated tracks (D_GA_) for the synthetic data set. (c) D_AG_ for the real data set, (d) D_GA_ for the real data set. High distances for the synthetic sets are due to low thresholds that interpret noise as neutrophils. The increase in D_GA_ in (d) is caused by higher levels that do not detect faint neutrophils, this in turn will reduce D_AG_ as with the higher threshold, the neutrophils which are detected are the brightest and thus the tracking is more precise.

**Figure 7:**
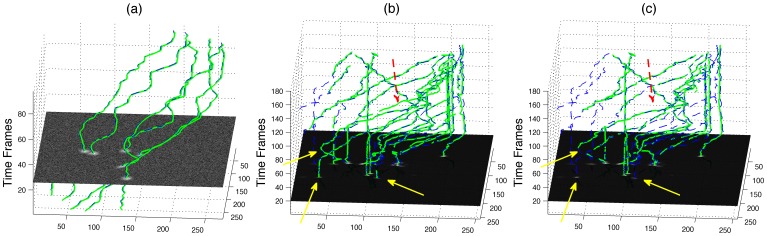
Comparison of automatic tracks against the gold standard. A synthetic data set is shown in (a) whilst (b,c) are real data sets. In (a,c) the thresholds are 140% of the automatically detected values, while for (b) they are 60% of the detected values. The automatic tracks are displayed as thick solid lines and the gold standard as thin dashed lines, and one slice of the intensity data sets is presented with the tracks. In the real data set, the high thresholds prevented the low intensity neutrophils from being detected (solid arrows) and therefore no tracks were generated for these neutrophils with corresponding high D_GA_. With lower thresholds, the faint neutrophils were detected, and other neutrophils were also tracked (dashed arrow), this track could have been generated by noise or could have been missed during the manual tracking. It should be noticed that where *PhagoSight* detected the neutrophils, the tracks are very close to the gold standard.

For both real and synthetic cases, we modified the thresholds from 40% to 140% from the automatically detected thresholds to test the robustness against variation of that input parameter. As expected, the errors increased toward the extreme values (Fig. 6). However, the results were fairly stable for the range of threshold values between 60% and 140%. In the synthetic sets, it was only high levels of noise that increased the distances; the errors arose from low thresholds that segmented noise as neutrophils incorrectly. For the real data sets, the thresholds between 80%*–*140% provided stable results of D_AG_. D_GA_ on the other hand, increased with the thresholds, which indicated that the manual tracking followed faint neutrophils, which were not detected by the algorithms. Fig. 7 illustrates both sets of tracks for one synthetic set with the threshold levels at 140% from the automatic levels (a), and one real set with thresholds at 60% (b) and 140% (c). Thick solid lines correspond to the automatic tracks and thin dashed lines to the gold standard. It is interesting to notice first, that the automatic tracks are very close to the manual tracks in both data sets when high thresholds were selected. Second, it can be observed that faint neutrophils were tracked manually (solid arrows) and are even difficult to see to a human observer. These neutrophils were not detected when the thresholds were raised and thus the error in D_GA_ increased. Third, when low thresholds were selected, besides the tracks corresponding to the faint neutrophils, there were other tracks that either correspond to noise or to a neutrophil that was not captured in the manual tracking (dashed arrows). As an indication of the computational complexity, the time to process one of the real data sets, from reading the images to producing the handles was approximately 20 seconds.

**Figure 8:**
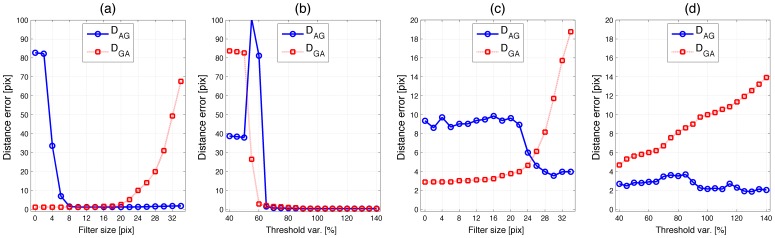
Comparison of the segmentation and tracking results against ICY for one synthetic and one real data set. In ICY, “size filter” was used with increasing values of size; *PhagoSight* input thresholds were automatically determined and then modified from 40% to 140% of the original. (a) Synthetic data set tracked with ICY, (b) Synthetic data set tracked with *PhagoSight*, (c) Real data set tracked with ICY, (d) Real data set tracked with *PhagoSight*. Solid line and circle markers corresponds to distance from the automatically generated tracks to the gold standard (D_AG_) and dotted line with square markers corresponds to the distance from the gold standard to the automatically generated tracks (D_GA_).

We compared the tracking results of *PhagoSight* against the open-source software ICY [29] for one synthetic data set (highest noise) (Fig. 8a,b) and one real data set (set number 6), (Fig. 8c,d). In ICY we used the plug-in “*Spot Detector*”, with “*Bright spots over dark background*” and filtering with “*Size Filtering*” with increasing sizes as noise had a very strong impact on the detection and subsequent tracking. Then we used the “*Probabilistic Particle Tracker*” plug-in with “*Single motion model*”, “*Brownian motion*” and “*Maximum likelihood association*”. We observed similar behaviours when modifying the size of the filter as the variation of the thresholds. A small filter that allows small objects to be segmented and tracked generated a large number of tracks; some of these tracks were close to the gold standard and thus D_GA_ was low but D_AG_ was high. As the size of the filter increased, the number of objects decreased; D_AG_ also decreased as D_GA_ increased. These trends were present in both synthetic and real data sets and the errors were comparable in both cases.

### b. Relationship between Neutrophil Volume and Recruitment to Sites of Tissue Injury

Cell volume has been suggested to be a measure of neutrophil activation [52] but it is difficult to assess changes in neutrophil volume during cell recruitment *in vivo*. Long unbroken tracks are required to relate the volume of individual cells to their longitudinal position. The advantages of using *PhagoSight* for volume analysis are (a) the hysteresis segmentation with post-processing to split large volumes formed by several cells either as static clumps of cells at the site of injury or due to a collision during migration, (b) the keyhole model of movement used for tracking instead of a nearest cell which is commonly used [22], together with the collision analysis that rendered more reliable tracks, (c) the proofreading tools which allow the manual correction of errors in tracking, and finally, (d) the removal of single random cells not part of any track, which would bias the measurements, i.e. a single object that appears at only one frame and then disappears is not taken into account for future calculations. We therefore measured the volume of migratory neutrophils whose tracks spanned more than 15 minutes (30 time points) and had an absolute velocity ≥ 0.68 µm/min (2 pixels/time point) using *PhagoSight*. These criteria allowed us to analyse neutrophils migrating towards the site of injury with long enough tracks to characterise the relationship between volume and proximity to the site of injury, and to exclude a confounding effect of groups of cells at the wound edge. The data were expressed as volume against relative position in the field of view. The field of view was split into 25 adjacent regions each containing 20 MATLAB® columns, with higher numbers denoting neutrophils closer to the wound region. The volume was averaged in each band and normalised to the mean of the data set for each larva, which were then pooled in the final analysis.

**Figure 9:**
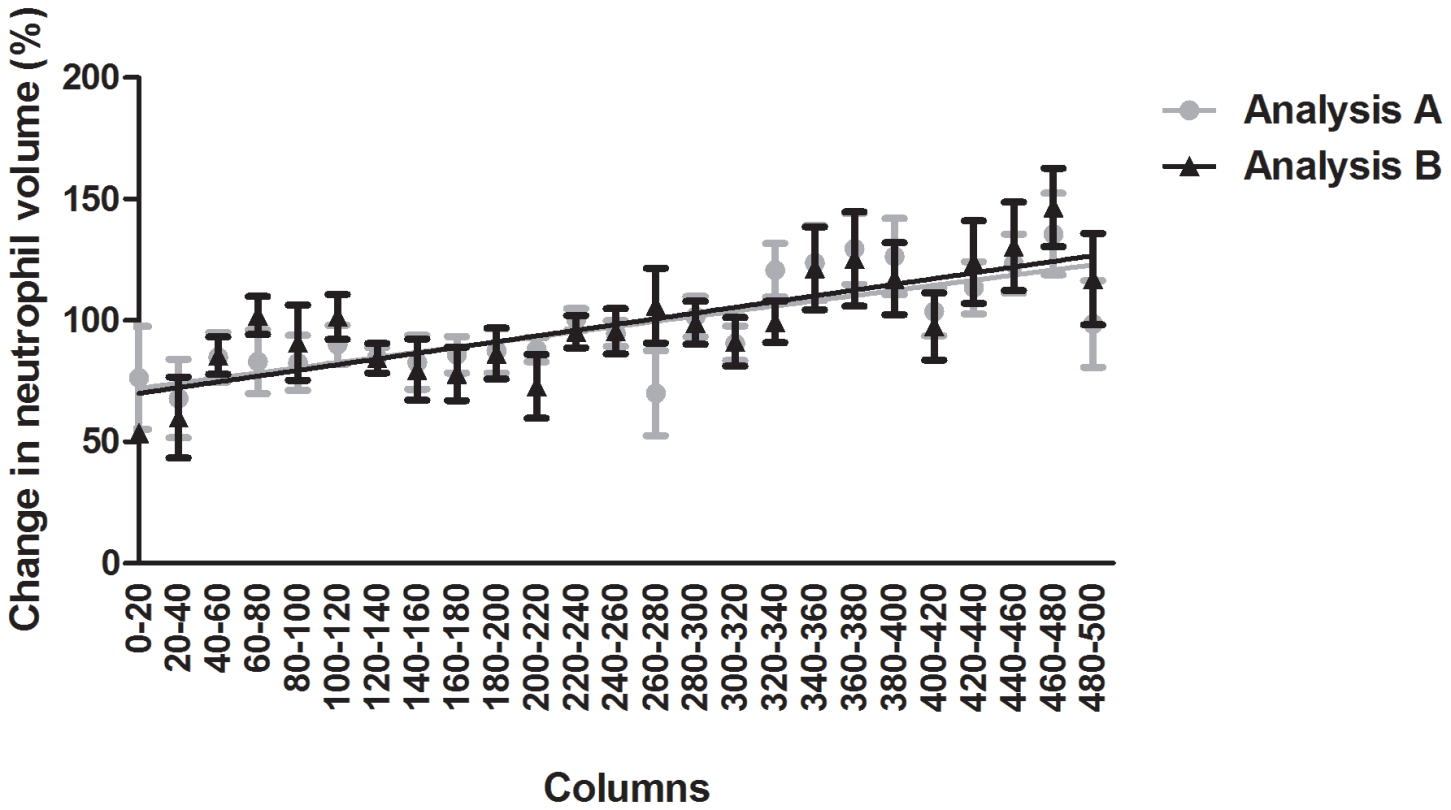
Mean neutrophil volume relative to its position in an area anterior to the wound region. The field of view was split into 25 adjacent regions each containing 20 MATLAB® columns, with higher numbers denoting neutrophils closer to the wound region. The volume was averaged in each band and normalised to the mean of the data set for each larva, which were then pooled in the final analysis. Two sets of thresholds were used. Analysis A uses higher thresholds than analysis B.

Neutrophil volume increased as neutrophils travelled towards the site of injury (Fig. 9). The increase was confirmed by linear regression analysis that showed the slope is significantly non-zero (n = 6, *P*<0.0001, r^2^ = 0.21). This strongly supports the hypothesis that neutrophil volume increases as neutrophils are recruited to the wound, and suggests that dynamic changes in volume analysed in this way might be a reliable measure of neutrophil activation. To investigate the sensitivity of the volume/position relationship to threshold levels, we ran the analysis with two sets of thresholds: a higher set (analysis A) and a lower set (analysis B). In both analyses, the volume of the neutrophils increased as they migrated towards the wound region (Fig. 9). Linear regression analysis showed that the slope for both analyses was significantly non-zero (n = 6, *P*<0.0001, r^2^ = 0.21).

## Discussion

Here we present *PhagoSight*, an open-source MATLAB® package of algorithms written for the analysis of immune cells as observed with confocal or multiphoton microscopes.

We used *PhagoSight* to demonstrate an increase in neutrophil volume when migrating towards a wound region *in vivo*. To our knowledge, this phenomenon has not previously been demonstrated from observations made directly *in vivo*, thus simultaneously demonstrating both the power of our zebrafish model and of *PhagoSight* to detect biological phenomena. The change in cell shape as neutrophils migrate towards the wound would only affect the volume measurement if there was a significant change in fluorescent intensity over time. This does not appear to be an issue in our data set, as if this were the case, cell volume would decrease towards the wound, in contrast to the increase in neutrophil volume that we observe. Previously, the volume of neutrophils has been measured using the mass of cells established by transmission electron microscopy [53] or using the diameter of neutrophils measured using a micropipette of known size [54]. Reconstruction of confocal sections of images of human neutrophils migrating through a collagen gel matrix have also been used to measure volume *in vitro* [55]. Despite being unable to calculate neutrophil volume accurately *in vivo* in the past, several studies *in vitro* have highlighted the importance of changes in volume for the migratory capacity of neutrophils [55], [56], [57].

Neutrophil volume increases due to an influx of water as the cell membranes extend to form pseudopods, which allow the neutrophil to migrate towards a site of injury or infection [55]. Rosengren *et al*. [55] demonstrated that human neutrophils increase in volume when exposed to a concentration gradient of a stimulating substance, but did not significantly increase in volume when stimulated by a single concentration of the same substance. Aquaporins have been shown to play a crucial role in this process. Karlsson *et al*. [57] demonstrated that phosphorylation of aquaporin 9 and its translocation to the cell membrane was necessary for the activation and migration of primary human neutrophils.

*PhagoSight*, like other algorithms, has limitations, for example: when a collision is detected between two neutrophils, the segmentation performed by *PhagoSight* is rather good as judged by a visual observation. However, when neutrophils start to accumulate at the wound, the collision may involve three or more neutrophils, and the higher the number of neutrophils, the higher the likelihood of performing an incorrect segmentation. A second limitation is related to the speed at which the cells move from time point to time point. When a cell jumps a distance several times its size, and, very importantly, there are other cells in the vicinity, it can also create errors in the tracks. For those cases, the possibility of manual intervention is useful as a user can perform track corrections. However, in most of the data sets that we tracked for this paper, both synthetic and biological, and other published results [44], [51], [58], *PhagoSight* provided satisfactory results with minimum human intervention. Another important issue to note, is that some of the measurements derived from the data sets are dependent on an arbitrarily defined “wound region”, a wider wound region could imply that more neutrophils enter the wound and the reverse would happen with a narrower region.

## Conclusion

We have developed *PhagoSight* for the tracking of immune cells in a zebrafish model, although there is broad applicability of these approaches. We have demonstrated the potential of our algorithm to detect parameters of biological significance and have described the additional parameters available in *PhagoSight* which may be of value in understanding the complex behaviour of immune cells in future studies.
